# Identification and Validation of Pyroptosis-Related Gene Signature to Predict Prognosis and Reveal Immune Infiltration in Hepatocellular Carcinoma

**DOI:** 10.3389/fcell.2021.748039

**Published:** 2021-11-08

**Authors:** Xiao-Wei Fu, Chun-Qing Song

**Affiliations:** ^1^ Fudan University, Shanghai, China; ^2^ Key Laboratory of Growth Regulation and Translational Research of Zhejiang Province, School of Life Sciences, Westlake University, Hangzhou, China; ^3^ Westlake Laboratory of Life Sciences and Biomedicine, Hangzhou, China; ^4^ Laboratory of Gene Therapeutic Biology, Institute of Basic Medical Sciences, Westlake Institute for Advanced Study, Hangzhou, China

**Keywords:** pyroptosis, prognosis, biomarker, immune infiltration, drug sensitivity, hepatocellular carcinoma

## Abstract

**Background:** Hepatocellular carcinoma (HCC) is characterized by a poor prognosis and accounts for the fourth common cause of cancer-related deaths. Recently, pyroptosis has been revealed to be involved in the progression of multiple cancers. However, the role of pyroptosis in the HCC prognosis remains elusive.

**Methods:** The clinical information and RNA-seq data of the HCC patients were collected from the TCGA-LIHC datasets, and the differential pyroptosis-related genes (PRG) were firstly explored. The univariate Cox regression and consensus clustering were applied to recognize the HCC subtypes. The prognostic PRGs were then submitted to the LASSO regression analysis to build a prognostic model in the TCGA training cohort. We further evaluated the predictive model in the TCGA test cohort and ICGC validation cohort (LIRI-JP). The accuracy of prediction was validated using the Kaplan—Meier (K-M) and receiver operating characteristic (ROC) analyses. The single-sample gene set enrichment analysis (ssGSEA) was used to determine the differential immune cell infiltrations and related pathways. Finally, the expression of the prognostic genes was validated by qRT-PCR *in vivo* and *in vitro*.

**Results:** We identified a total of 26 differential PRGs, among which three PRGs comprising GSDME, GPX4, and SCAF11 were subsequently chosen for constructing a prognostic model. This model significantly distinguished the HCC patients with different survival years in the TCGA training, test, and ICGC validation cohorts. The risk score of this model was confirmed as an independent prognostic factor. A nomogram was generated indicating the survival years for each HCC patient. The ssGSEA demonstrated several tumor-infiltrating immune cells to be remarkably associated with the risk scores. The qRT-PCR results also showed the apparent dysregulation of PRGs in HCC. Finally, the drug sensitivity was analyzed, indicating that Lenvatinib might impact the progression of HCC via targeting GSDME, which was also validated in human Huh7 cells.

**Conclusion:** The PRG signature comprised of GSDME, GPX4, and SCAF11 can serve as an independent prognostic factor for HCC patients, which would provide further evidence for more clinical and functional studies.

## Introduction

Statistics indicate that by 2025, there will be more than 1 million new cases of liver cancer annually, posing a significant challenge for the global medical field (Llovet et al., 2021). The HCC ranks as the fourth leading cause of cancer-related mortality accounting for 90% of liver cancer cases (Llovet et al., 2016). Recent studies indicated the 5-years survival rate of HCC patients to be lessened by 20% globally and as low as 12% in Asian countries (Craig et al., 2020). Epidemiological studies reported the hepatitis B virus, hepatitis C virus, alcoholism, and aflatoxin as common inducing factors of HCC (Yang et al., 2019).

HCC develops rapidly as well as stealthily such that the patients are diagnosed only when the disease has progressed to the middle and late stage. Alpha-fetoprotein (AFP) is currently a gold standard for the diagnosis and prognosis of HCC. However, its sensitivity and specificity are not very high owing to the interference in the expression by many non-HCC related factors (Cai et al., 2019). In addition, the accuracy of prognosis is also affected by the heterogeneity of HCC (Liu et al., 2016). Therefore, it is urgent to develop a novel prognostic model to improve the accuracy of the prognostic judgment for HCC patients.

Cell death is one of the critical aspects of anti-tumor drug research. It may involve various patterns, including pyroptosis, apoptosis, necrosis, necroptosis, and ferroptosis (Shojaie et al., 2020). Pyroptosis is a novel lytic and pro-inflammatory programmed cell death. It is mediated by the cysteine aspartate specific protein kinases (caspases) 1, 4, 5, and 11 (Galluzzi et al., 2018), and the final step is dependent on the activity of the gasdermin (GSDM) family proteins to form pores in the cell membrane (Kovacs and Miao, 2017; Humphries et al., 2020). Pyroptosis is morphologically characterized by cell swelling, plasma membrane permeability, and the gradual release of the inflammatory factors (Rathinam and Fitzgerald, 2016), while necrosis makes the cytoplasmic membrane rupture with a blast. Pyroptosis is also different from apoptosis because apoptosis does not induce cell membrane breakdown and inflammatory response (Bertheloot et al., 2021). In addition, pyroptosis differs from necroptosis, another inflammatory programmed cell death executed by the mixed lineage kinase domain-like protein (MLKL), in that pyroptosis maintains mitochondria integrity but necroptosis does not (Bertheloot et al., 2021). Furthermore, pyroptosis differs from ferroptosis because ferroptosis is featured by iron-dependent oxidative perturbations, increased membrane density, and small mitochondria (Sun et al., 2021). As studies continue, pyroptosis has been found to plays a vital role in tumor formation and development. Due to its dual functions of resisting infection and inducing pathological inflammation, pyroptosis has dual roles in promoting tumors and changing tumor immune microenvironments (Kong et al., 2015; Rathinam and Fitzgerald, 2016; Xia et al., 2019). Specifically, the role of pyroptosis in the HCC development and prognosis remains elusive.

This study systematically analyzed the differential expressions of PRGs between the HCC and normal samples; explored the clinical prognostic value of these genes through Cox expression analysis; established an independent prognostic model based on PRGs; investigated the relationship between the pyroptosis and tumor immune microenvironments; validated the mRNA expressions of PRGs *in vivo* and *in vitro*; and evaluated the drug sensitivity of these prognostic factors. Therefore, this study provides potential targets for the prognosis and treatment of HCC patients.

## Materials and Methods

### Data Acquisition

The RNA sequence data and related clinical information of 374 liver cancer patients (TCGA-LIHC) were acquired from the TCGA website (https://portal.gdc.cancer.gov/repository). The gene expression data were normalized by scale method using the “limma” package (Yuan C. et al., 2021). After excluding the missing clinical information of patients, 370 HCC patients were randomly separated into the training and the test groups by the “caret” package. Besides, transcriptomics data with clinical features of 231 HCC patients (LIRI-JP) were downloaded from the ICGA database (https://dcc.icgc.org/projects/LIRI-JP). These HCC patients were HBV or HCV carriers from Japan. The gene read count values of these patients were also normalized, and both the TCGA and ICGC data were public.

### Analysis of Differential PRGs

A total of thirty-three PRGs were retrieved from the previous literature and are listed in Supplementary Table S1 (Man and Kanneganti, 2015; Tang et al., 2020; Ye J. et al., 2021). The differentially expressed genes (DEGs) were analyzed by the “limma” package in the R software and visualized by the heatmap and volcano plot with adjusted *p*-value < 0.05 in the TCGA cohort. In addition, a protein-protein interaction network for the PRGs was generated using the software GENEMANIA (http://genemania.org/).

### Establishment and Validation of the Prognostic Model Based on the PRGs

The Univariate Cox analysis was employed to screen the PRGs with the prognostic value, and the cut-off *p*-value was set at 0.05, and 17 survival-related genes were used for further study. The prognostic model was established to minimize overfitting using the LASSO-penalized Cox regression analysis via the “glmnet” R package (Du et al., 2021). Eventually, the three genes and their coefficients were retained, and the minimum criteria determined the penalty parameter (λ). The risk score was obtained using the formula: risk score = (βA × Gene A expression) + (βB × Gene B expression) ⋯ + (βN × Gene N expression), in which β represents regression coefficient (Yang et al., 2021). The HCC patients were separated into the high- and low-risk groups based on the median risk score. Then, the principal component analysis (PCA) for the two risk groups was constructed using the “limma” and “scatterplot3d” R packages in terms of gene expressions in the prognostic model (Yuan M. et al., 2021). The survival analysis between the two risk groups was carried out using the “survminer” R package, and the ROC curve analysis was performed via the “survival” and “timeROC” R packages (Fang et al., 2021). Besides, the univariate and multivariate Cox regression was utilized to determine the independent prognostic value of the 3-gene signature.

To validate the efficiency of our model, the patients in the TCGA internal test cohort or ICGC external validation cohort (LIRI-JP) were applied. The mRNA levels of PRGs were normalized according to the “scale” function, and the risk score was calculated using the same formula applied in the TCGA training cohort. The TCGA test or ICGC cohort patients were separated into the low- and high-risk groups using the median risk score obtained from the TCGA training cohort.

### Development of a Predictive Nomogram

Based on the risk score and different clinical features (gender, age, histologic grade, and pathological stage), a nomogram model was established to predict the survival years for the HCC patients using the “rms” and “survival” packages (Dai et al., 2021).

### Gene Set Enrichment Analyses

The GSEA analysis was carried out between the two risk groups using the GSEA software 4.0.1 to identify the differential KEGG pathways. The normalized enrichment scores and nominal *p*-values were determined for analyzing the enrichment levels and statistical significance. In addition, the infiltrating scores of the 16 immune cells and activities of 13 immune-related pathways were analyzed using ssGSEA provided in the “GSVA” R package (Zhao et al., 2021).

### Drug Sensitivity

The NCI-60 dataset covering nine cancer categories was available on the CellMiner homepage (https://discover.nci.nih.gov/cellminer) (Shankavaram et al., 2007; Shankavaram et al., 2009). In addition, Pearson correlation analysis was performed to indicate the relevance between the independent prognostic PRGs and drug sensitivity. Drugs used in this sensitivity analysis are those approved by the FDA or those in clinical tests.

### Construction of the Hepatocellular Carcinoma Mouse Model

The *FVB* mice (8 weeks) were purchased from Westlake University (Zhejiang, China). Plasmids for injection were harvested using the Endotoxin Free Maxiprep kit (Qiagen) and delivered to the *FVB* mice as a mixture using the hydrodynamic tail vein injection for HCC formation as described before (Song et al., 2017). Dosages of these plasmids were: px330-U6-sgP53 (mouse) 20 μg, pT3-EF1α-c-Myc (human) 5 μg, pCMV-sleeping beauty transposase 2 μg. The daily abdominal palpation of mice was observed, and the mice were then sacrificed when they suffered from high burdens of liver tumors. The mice were raised according to the protocols approved by the Institutional Animal Care and Use Committee of Westlake University. The researcher followed the standard biosecurity and institutional safety procedures in this study.

### Cell Culture and Drug Treatment

The human normal liver cell line HL-7702 and HCC cell lines (SK-Hep1 and Huh7) were cultured in a DMEM medium containing 10% FBS in a 5% CO_2_ incubator at 37°C. In addition, huh7 cells were treated by Lenvatinib (HY-10981, MCE) with different concentrations (0, 0.5, 1, 5, 10 μM) for 48 h based on the previous study (Jin et al., 2021).

RNA extraction and quantitative real-time reverse transcriptase-polymerase chain reaction (qRT-PCR).

The total RNA in the liver tissues or liver cell lines was extracted using the TRIZOL (Invitrogen) reagent. Then, the cDNA was obtained by reverse transcription using the cDNA Synthesis Mix (Novoprotein, E047-01B) and analyzed using quantitative PCR (Novoprotein, E096-01B). Finally, the mRNA expression levels were normalized with the *ACTB* gene. All the primers used in this study are listed in Supplementary Table S2.

### Western Blotting

The total proteins in Huh7 cells treated with Lenvatinib were extracted by RIPA buffer containing 1% PMSF (Beyotime) and quantified by the BCA method (Beyotime). Twenty microgram proteins were used for western blotting against GSDME (1:2000, ab215191, Abcam) and Hsp90 (1:2000, 610419, BD Bioscience).

### Statistical Analysis

The one-way ANOVA was adopted to compare the DEGs between the HCC and normal liver tissues. The Mann—Whitney test using the BH method adjusted *p*-value was adopted to measure the ssGSEA scores. The entire statistical analyses were achieved using the R software 4.0.1. The statistical significance was considered as a *P* -value less than 0.05 if not otherwise specified.

## Results

### Differential Expression of PRGs Between the Normal and Tumor Tissues

**FIGURE 1:**
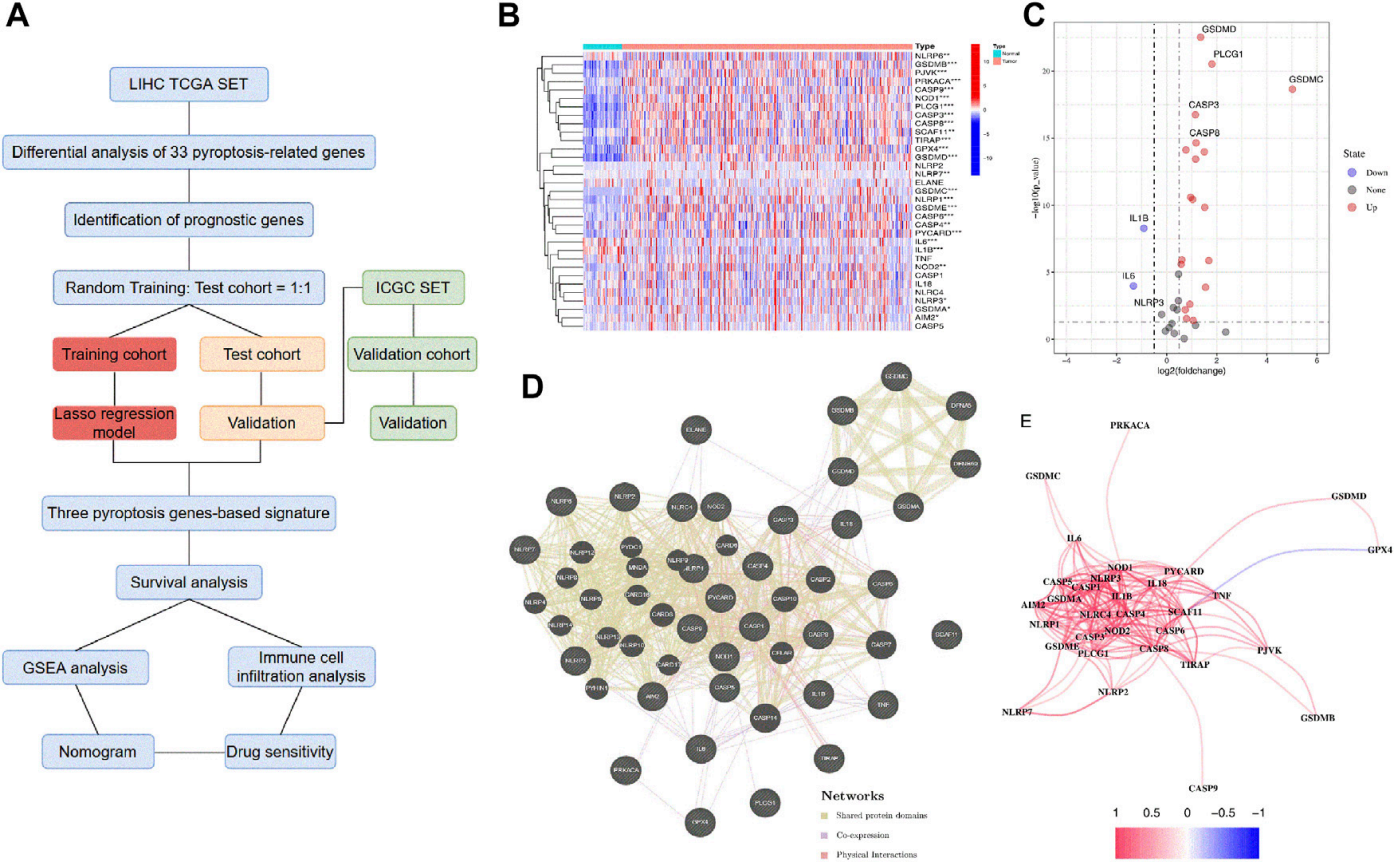
Screening of the PRGs in the HCC patients from the TCGA database. **(A)** Flow chart of the study. **(B)** Heatmap of the differential gene expression between the normal and the tumor tissues. **(C)** Volcano plot of the differentially expressed PRGs. **(D)** PPI network of the interactions among PRGs. **(E)** The correlation network (Redline: positive correlation; Blueline: negative correlation. The depth of the colors reflects the strength of the relevance).

We performed the differential expression analysis of 33 PRGs involved between the normal liver and HCC samples in the TCGA database (Figure 1A). The 33 PRGs were chosen based on their roles in pyroptosis according to previous studies (Karki and Kanneganti, 2019; Ye Y. et al., 2021; Ju et al., 2021). The results were presented in the heatmaps and volcano plots (Figures 1B,C). We identified 26 differential genes (*p* < 0.05), among which the three genes were significantly downregulated in the HCC samples, including *IL6*, *IL-1β*, and *NLRP3*. Conversely, another 23 genes were significantly upregulated, including *GSDMC*, *PLCG1*, *PYCARD*, *GSDME*, *NLRP1*, *GSDMB*, *GSDMD*, *CASP8*, *NOD1*, *CASP8*, *NOD1*, *CASP3*, *GSDMA*, *PJVK*, *TIRAP*, *NOD2*, *AIM2*, *GPX4*, *NLRP7*, *CASP9*, *CASP6*, *PRKACA*, *SCAF11*, *CASP4*, and *NLRP6*. The interactions within these PRGs were identified using GeneMANIA and constituted three interconnected subnetworks: the NLR superfamily regulatory network, the caspase family regulatory network, and the GSDM family regulatory network (Figure 1D). Finally, the correlation for these PRGs was analyzed and indicated in Figure 1E.

### Tumor Classification Based on the Prognostic Pyroptosis Regulators

**FIGURE 2:**
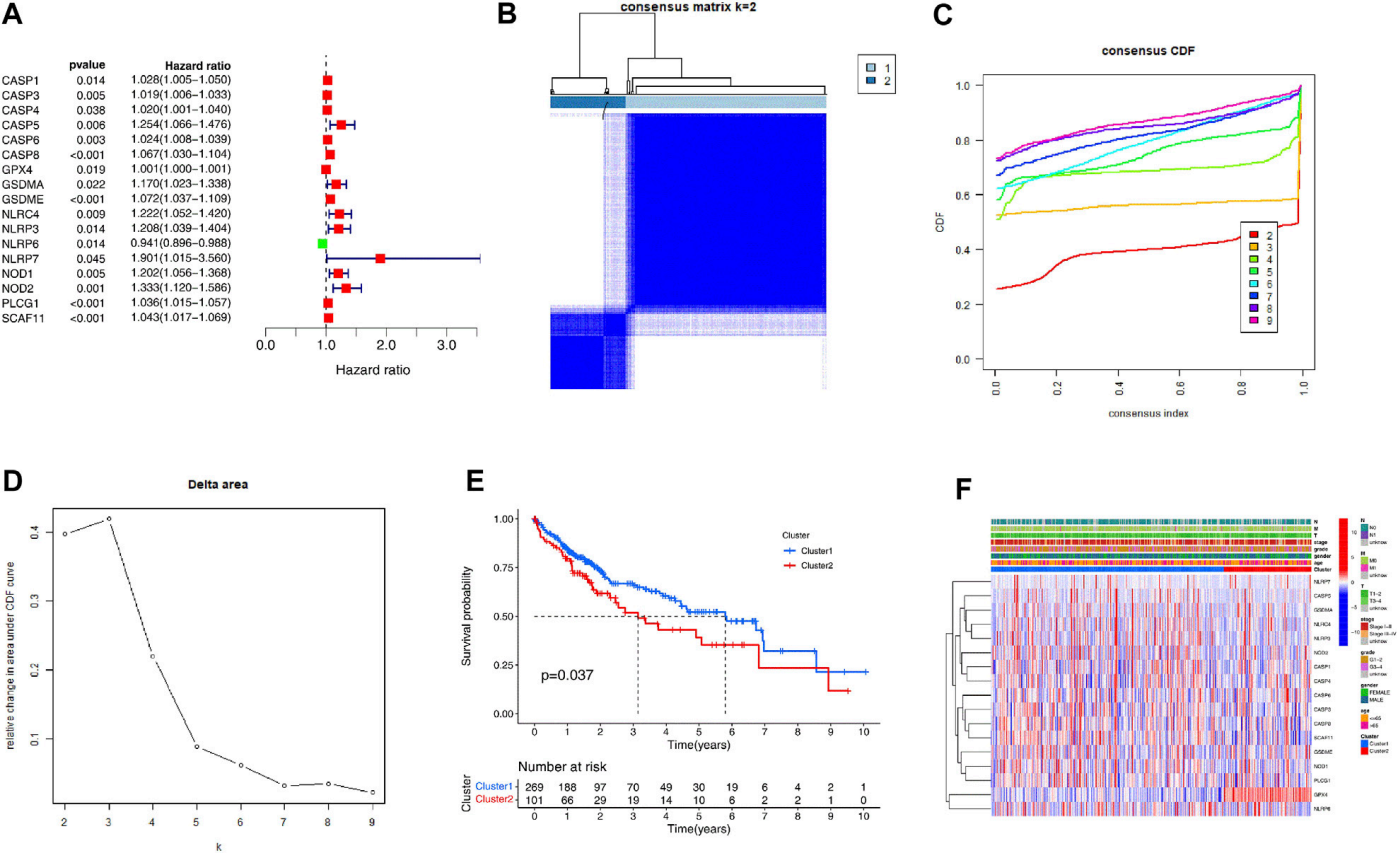
Identification of the molecular subtypes of the HCC patients using the PRGs associated with prognosis. **(A)** The univariate Cox Regression Analysis based forest plot in PRGs. **(B)** The HCC patients were stratified into 2 clusters based on the consensus clustering matrix (k = 2). **(C, D)** Consensus clustering model with cumulative distribution function (CDF) by k from 2 to 9. **(E)** The Kaplan—Meier curves (KM) of the overall survival in the two HCC clusters. **(F)** Heatmap with the correlation between the two groups and their clinicopathologic characters.

We first analyzed the prognostic values of PRGs in the TCGA cohort with the Univariate Cox regression analysis. The high expression of *CASP1*, *CASP3*, *CASP5*, *CASP6*, *CASP8*, *GPX4*, *GSDMA*, *GSDME*, *NLRC4*, *NLRP3*, *NLRP7*, *NOD1*, *NOD2*, *PLCG1*, and SCAF11 correlated with the poor survival of the HCC patients, as indicated by the Hazard ratio (HR) > 1 (Figure 2A). Then, we performed the consensus clustering analysis to investigate the relationship between these prognostic genes and HCC subtypes. According to the CDF value, we classified the 370 HCC patients into two clusters (k = 2, Figures 2B–D), and we found that the patients from cluster 1 tended to survive longer than the patients from cluster 2 (Figure 2E), implying a significant prognostic value of these PRGs. Furthermore, the two clusters did not differ in the clinical parameters such as stage, grade, gender, age, and TMN (Figure 2F).

### Establishment of an Independent Prognostic Risk Model in the TCGA Training Cohort

**FIGURE 3:**
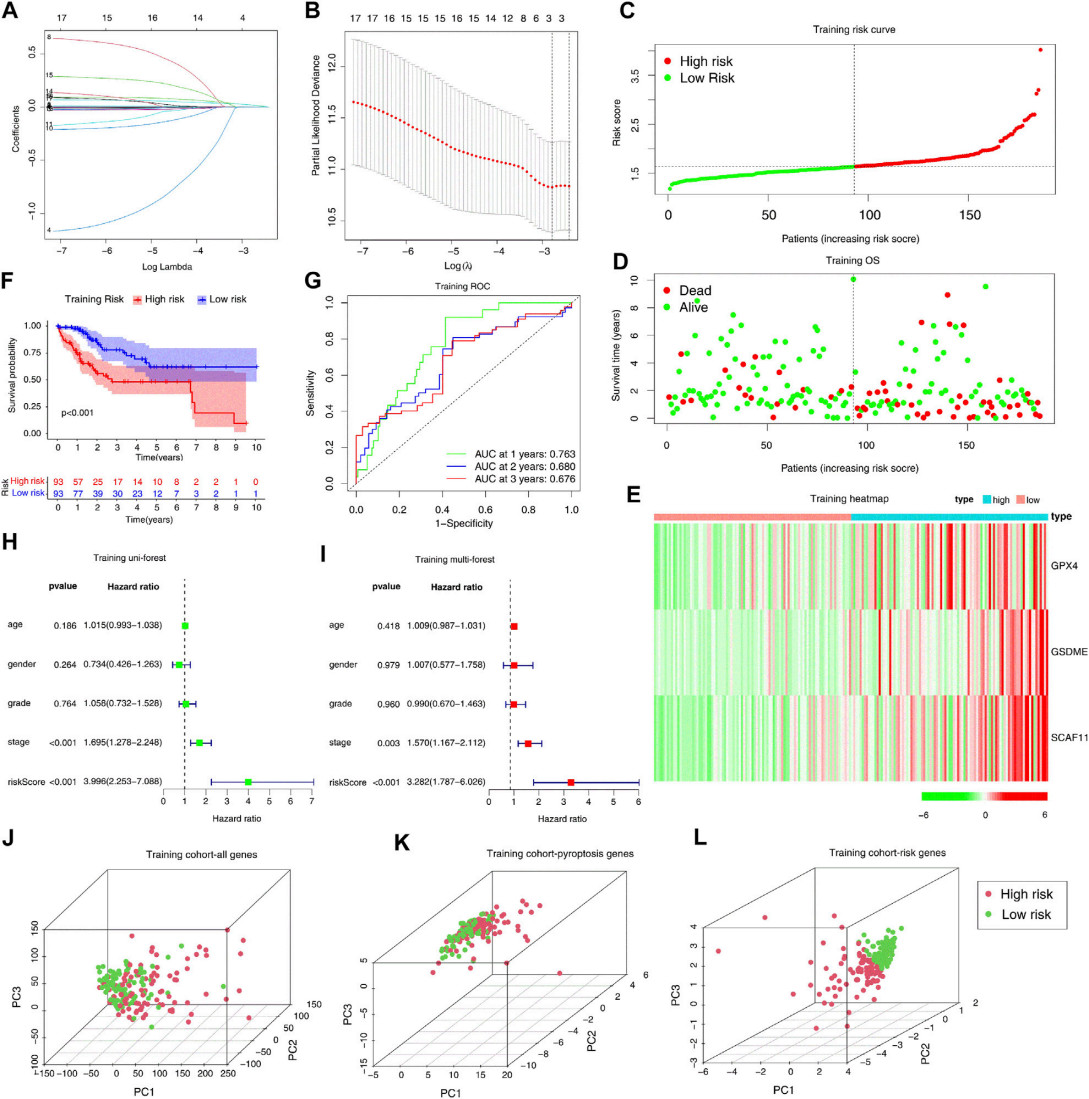
Construction of the pyroptosis genes-based independent prognostic model in the TCGA training cohort. **(A, B)** Construction of the LASSO regression model based on the 17 predictive genes in the TCGA training cohort. **(C–E)** Distribution of the risk scores, survival status, and expression of the three pyroptosis-related risk genes. **(F)** The KM analysis of the overall survival in the high-risk and low-risk groups. **(G)** The ROC analysis to evaluate the predictive efficiency. **(H)** The univariate Cox analysis to assess the independence of three pyroptosis-related risk genes. **(I)** Multivariate Cox analysis to assess the independence of three pyroptosis-related risk genes. **(J–L)** PCA analysis of the high-risk and low-risk groups based on all genes, pyroptosis genes, and three risk genes.

We split the samples in the TCGA-LIHC dataset into two equal cohorts: training cohort and test cohort at random. We found no significant difference between the training and test cohorts from TCGA-LIHC in the leading clinical indicators (Supplementary Table S3). We performed lasso regression analysis using 17 prognostic genes in the training cohort to build the prognostic model. According to the minimum criteria, a risk model consisting of GSDME, GPX4, and SCAF11 was built (Figures 3A,B). The risk score was obtained by the formula: risk score = (0.0182*GSDME exp.) + (0.0005*GPX4 exp.) + (0.0188*SCAF11 exp.)

We separated the patients in the training cohort into the high- and low-risk groups based on the median risk score (Figure 3C). The high-risk group had more deaths and shorter survival years (Figure 3D). The heatmap analysis indicated that high-risk patients had increased expression levels of three risk genes (Figure 3E). In addition, the Kaplan-Meier curve indicated that the high-risk patients had worse overall survival (OS) than low-risk patients (*p* < 0.001). The subsequent ROC analysis demonstrated that this 3-gene risk model could robustly evaluate and predict the survival of the HCC patients (AUC = 0.763, Figure 3G). Furthermore, the univariate and multivariate Cox regression analyses determined whether the risk score derived from the prognostic risk model could act as an independent prognostic indicator. In the univariate Cox regression analysis, the risk score (*p* < 0.001, HR = 3.996, 95% CI: 2.253–7.088, Figure 3H) was a potential hazard factor. The multivariate analysis also indicated that the risk score could serve as an independent prognostic factor (*p* < 0.001, HR = 3.282, 95% CI: 1.787–6.026, Figure 3I). Finally, the PCA plot indicated that the high- and low-risk groups could be well-separated with the three risk genes, but not all the genes or all the pyroptosis genes (Figures 3J–L).

### Internal and External Validation of the Risk Signature

**FIGURE 4:**
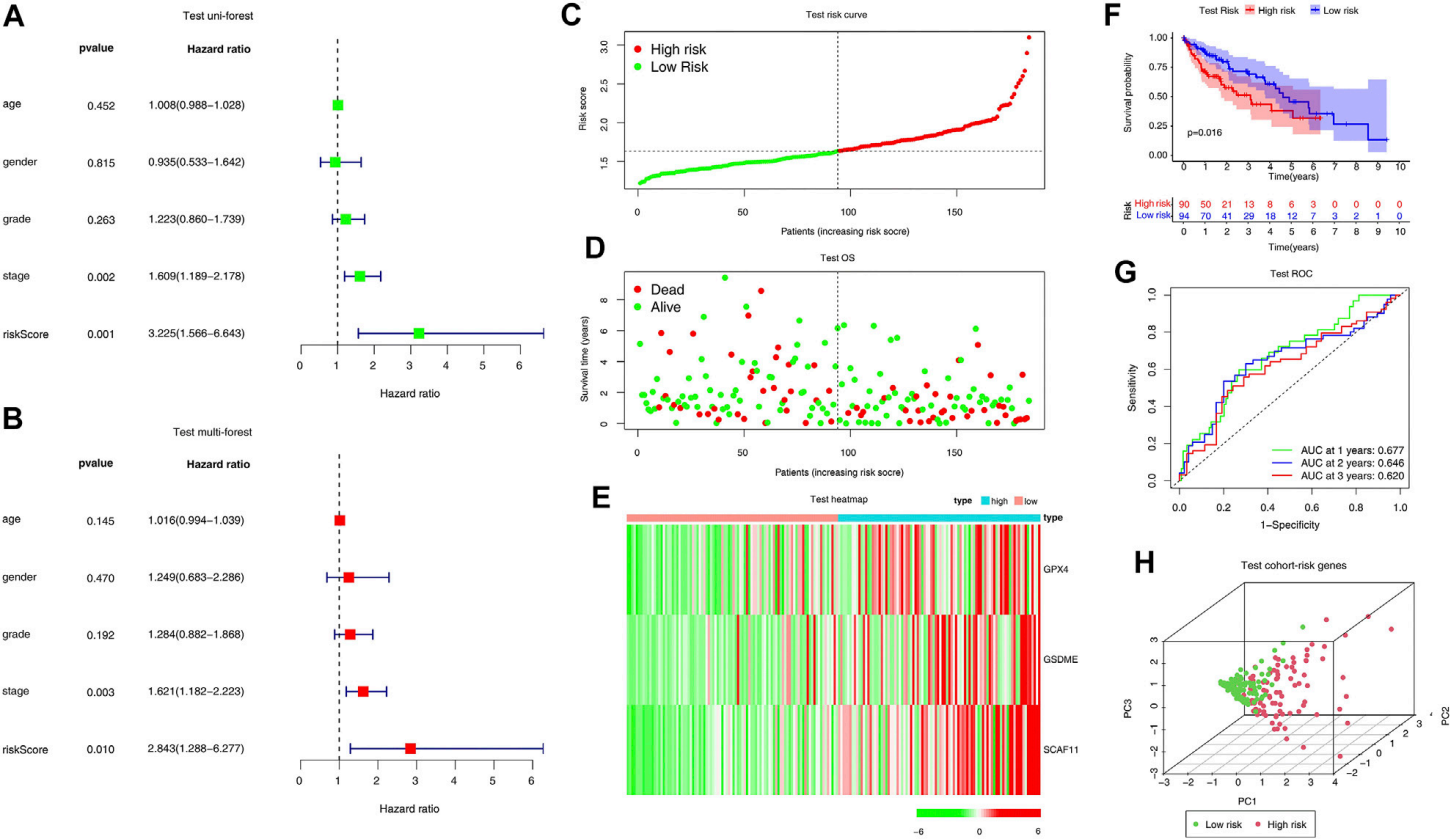
Internal validation of the risk model in the TCGA test cohort. **(A, B)** Forest plots of Univariate Cox analysis and Multivariate Cox analysis. **(C–E)** Distribution of risk scores, survival status, and expression of the three pyroptosis-related risk genes. **(F)** The KM analysis of the overall survival in the high-risk and low-risk groups. **(G)** The ROC analysis to estimate the predictive efficiency. (E) Univariate analysis. **(H)** PCA analysis of the high-risk and low-risk groups based on the three risk genes.

The efficiency of the risk model was validated in a TCGA internal test cohort which included 184 patients and an ICGC external validation cohort. In the TCGA internal test cohort, we divided patients into high or low-risk groups according to the median risk score calculated by the formula in the training cohort. The risk score is an independent prognostic factor in the test cohort as indicated by univariate and multivariate Cox regression analysis (*p* = 0.001, HR = 3.225, 95% CI = 1.566–6.643 for univariate, Figure 4A; *p* = 0.01, HR = 2.843, 95% CI = 1.288–6.277 for multivariate, Figure 4B). The high-risk group tended to have more deaths and higher expression of risk genes (Figures 4C–E). The KM curve indicated that overall survival was lower in the high-risk group (*p* = 0.015, Figure 4F).

**FIGURE 5:**
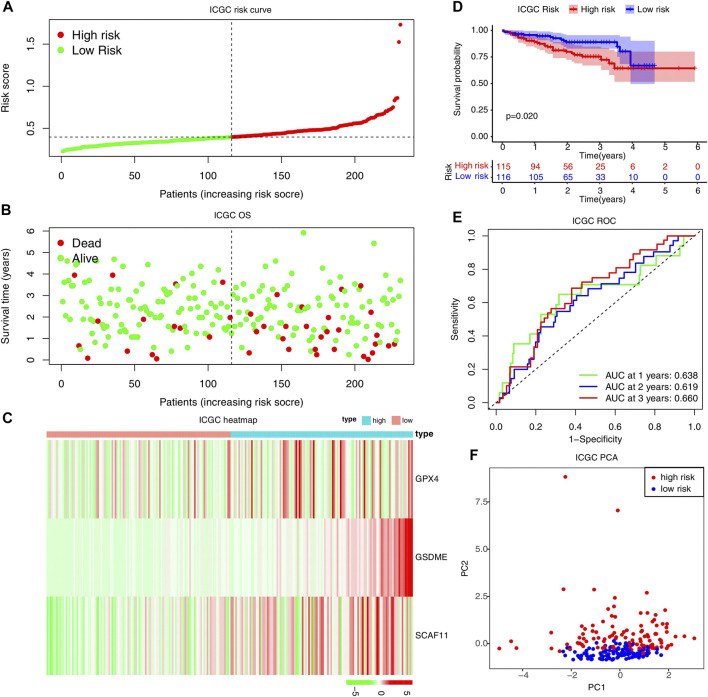
External validation of the risk model in the ICGC cohort. **(A–C)** Distribution of the risk scores, survival status, and expression of the three pyroptosis-related risk genes in the ICGC cohort. **(D)** The KM analysis of the overall survival in the high-risk and low-risk groups. **(E)** The ROC analysis to estimate the predictive efficiency. **(F)** The PCA analysis of the high-risk and low-risk groups based on the three risk genes.

Moreover, the ROC curve suggested that the model exhibited an excellent predictive capability (AUC = 0.677, Figure 4G). PCA analysis indicated that the expression levels of the 3-risk gene could separate high-risk patients from low-risk in the TCGA test cohort (Figure 4H). We split the ICGC external validation cohort into the high- and low-risk groups based on the risk score (Figure 5A). The low-risk group had fewer deaths and lower expression of the risk genes (Figures 5B,C). The KM curve and ROC analysis suggested that the overall survival of the low-risk group was higher (*p* = 0.02, Figure 5D), and the model was reliable (AUC = 0.638, Figure 5E). Finally, the PCA plot indicated that the risk genes were well able to separate the two risk groups (Figure 5F).

### Stratification Analysis of the Independent Prognostic Signature

**FIGURE 6:**
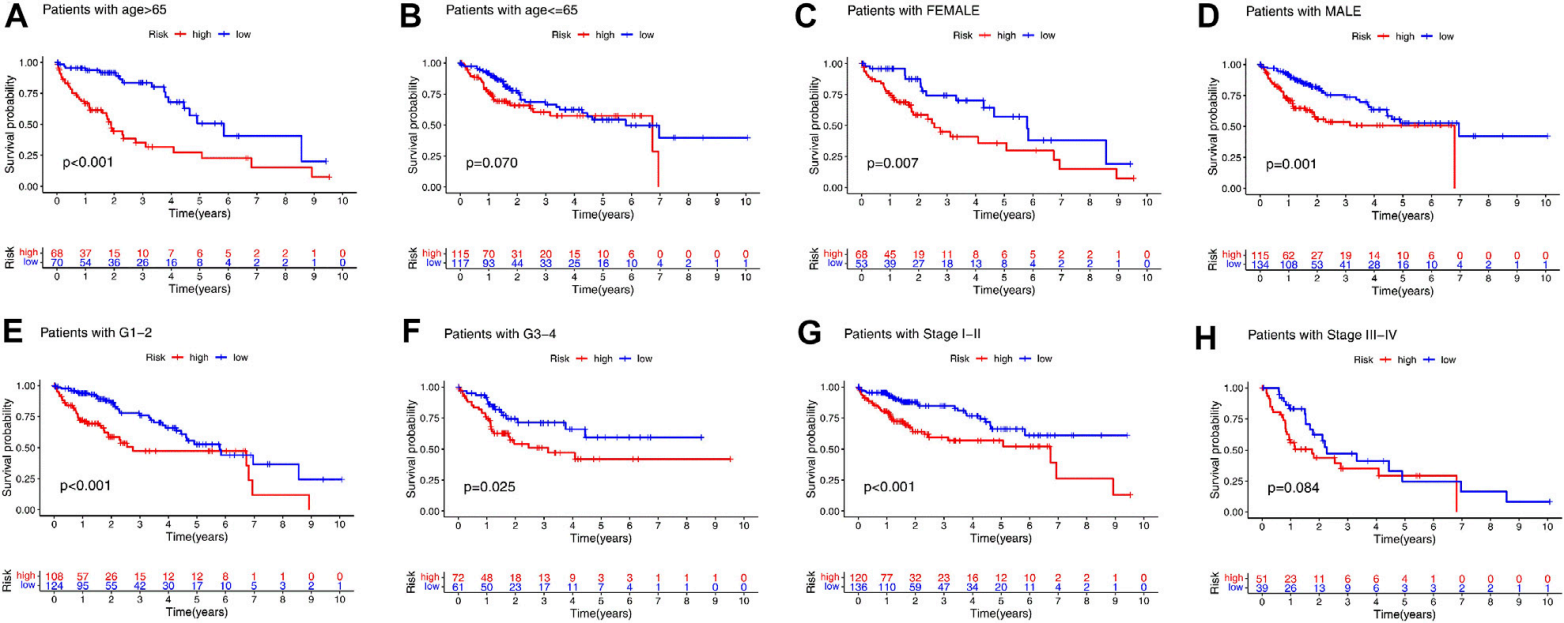
Identification of the HCC patients suitable for the risk model in the TCGA cohort. KM analysis of the overall survival in the high-risk and low-risk groups based on age **(A, B)**, gender **(C, D)**, grade **(E, F)**, and stage **(G, H)**.

We separated the patients in the TCGA-LIHC dataset into several subgroups according to the different clinical parameters. First, we investigated whether the high- and low-risk patients determined differences in survival. The clinical stratifications for the study included age (>65 vs ≤ 65), gender (female vs male), tumor grade (G3/4 vs G1/2), and AJCC stage (I/II vs III/IV). The KM curve showed that the high-risk patients had a poorer survival probability than the low-risk patients under the condition of age >65, female, male, G1–G2, G3–G4, or Stage I–II, except for age ≤65 or stage III-IV (Figures 6A–H).

### Establishment of a Prognostic Nomogram for Hepatocellular Carcinoma

**FIGURE 7:**
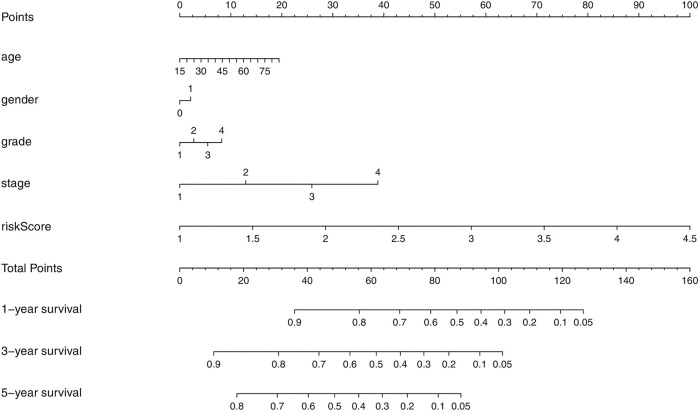
Nomogram containing the risk score to predict the overall survival in the HCC patients.

We developed a novel prognostic nomogram to offer a reliable and quantifiable method for predicting the survival of the HCC patients based on the risk scores and clinical features, such as age, gender, grade, and stage (Figure 7). The nomogram could effectively predict the probability of the 1, 3, and 5-years overall survival in the HCC patients.

### Gene Set Enrichment Analysis and Immune Activity Between the Subgroups

**FIGURE 8:**
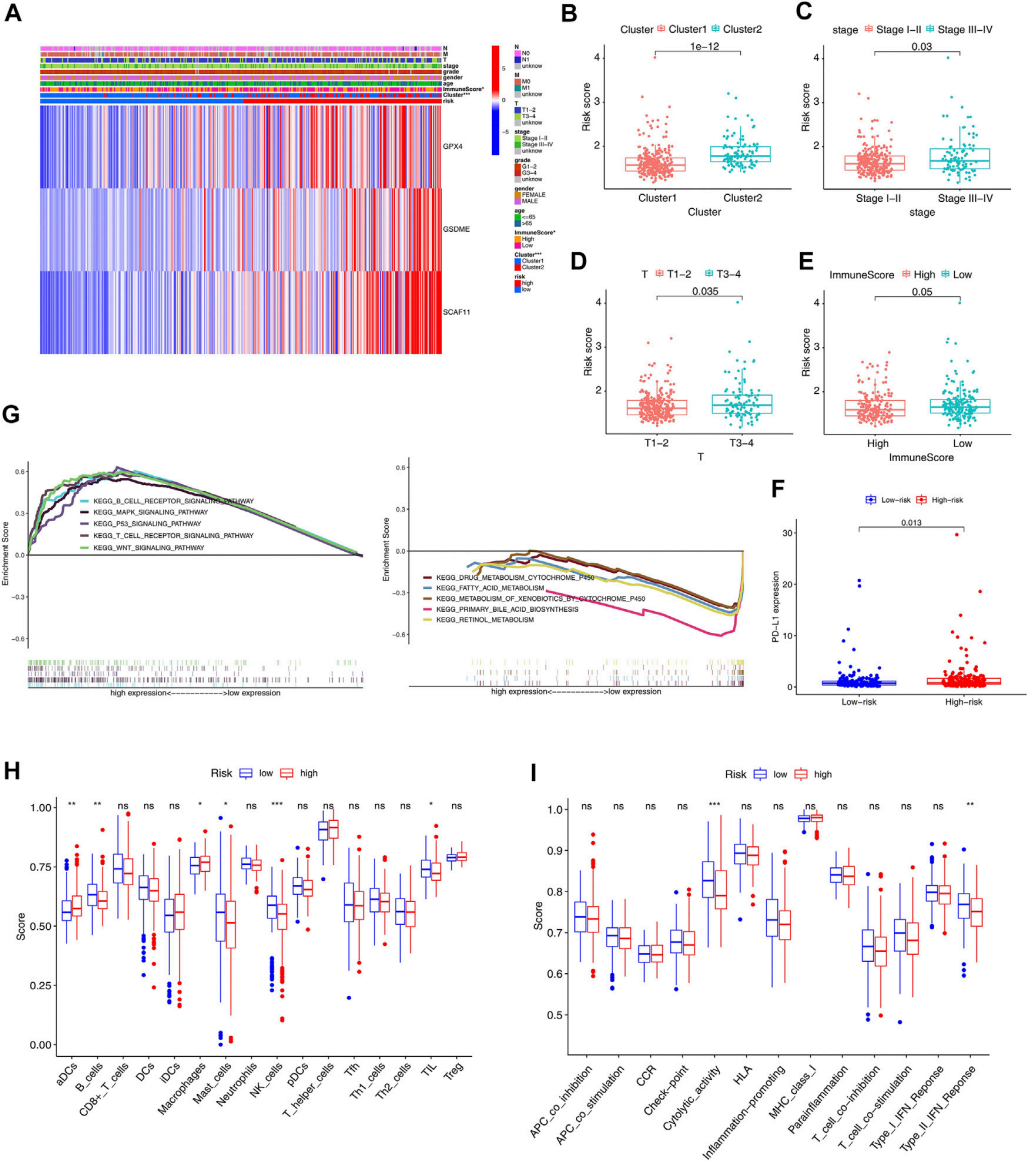
GSEA and immune correlation analysis of the immune cells and related immune pathways in the TCGA cohort. **(A)** Heatmap with correlations between clinical features and risk groups (*p < 0.05). For T, M, N, stage, and grade, the higher the numbers, the more advanced the tumors. For cluster, cluster 1 and cluster 2 were divided based on consensus clustering model. The risk and ImmuneScore were divided into high and low based on the median of all patients. **(B–E)** Risk scores of the HCC patients are classified by cluster, stage, Tstage, and immune scores. **(F)** PD-L1 expression in the low-risk and high-risk groups. **(G)** GSEA of the significantly enriched KEGG pathways in the TCGA cohort classified by independent prognostic genes. **(H, I)** The ssGSEA scores of 16 kinds of immune cells and 13 immune pathways (*p < 0.05, **p < 0.01, ***p < 0.001).

Given clinical information, the high- and low-risk groups were significantly correlated with the immune scores (Figure 8A). Specifically, the patients with lower immune scores favored high-risk scores compared to those with higher immune scores. In addition, the patients with higher stages picked higher risk scores (Figures 8B–E). Interestingly, the high-risk group had a high expression level of PD-L1 (Figure 8F). Furthermore, the GSEA analysis significantly enriched the immune-related signaling pathways, such as the B cell and T cell receptor signaling pathways (Figure 8G).

Further, the ssGSEA analyzed the differences in 16 kinds of immune cell infiltrations and 13 types of immune signal pathways. The high-risk group had decreased infiltrations of the B cells, mast cells, NK cells, and TIL and increased infiltrations of the DCS and macrophage compared to the low-risk group (*p* < 0.05, Figure 8H). Besides, the high-risk group exhibited suppression in the immune pathways, including the cytolytic activity and type II IFN response (*p* < 0.05, Figure 8I).

### Validation of the Differential Expression of the Independent Prognostic Genes

**FIGURE 9:**
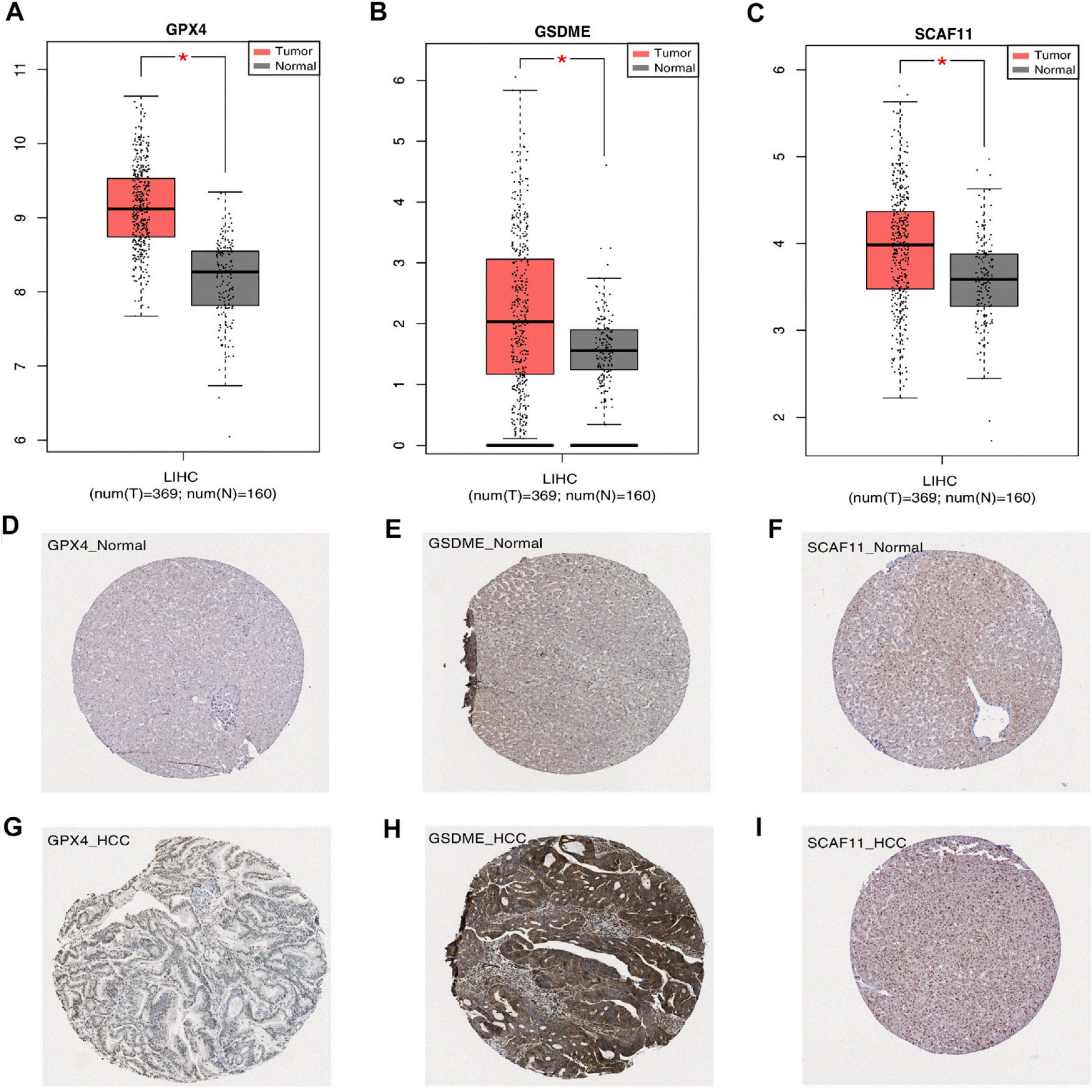
Expression of the independent prognostic genes. **(A–C)** The gene mRNA expressions of GPX4, GSDME, SCAF11 in the normal and tumor groups (*p < 0.05). **(D–I)** Immunohistochemistry of the GPX4, GSDME, SCAF11 in the normal and tumor groups from the HPA database.

We analyzed the differential mRNA levels of the three independent prognostic genes in the normal liver tissues (from TCGA and GTEx) and HCC tissues (from TCGA) by GEPIA. The HCC tissues favored an increased mRNA level of GSDME, GPX4, and SCAF11 (Figures 9A–C). In addition, we examined the protein levels of these three genes using the HPA database. The results showed that the HCC tissues had higher protein levels of GSDME, GPX4, and SCAF11 (Figures 9D–I).

**FIGURE 10:**
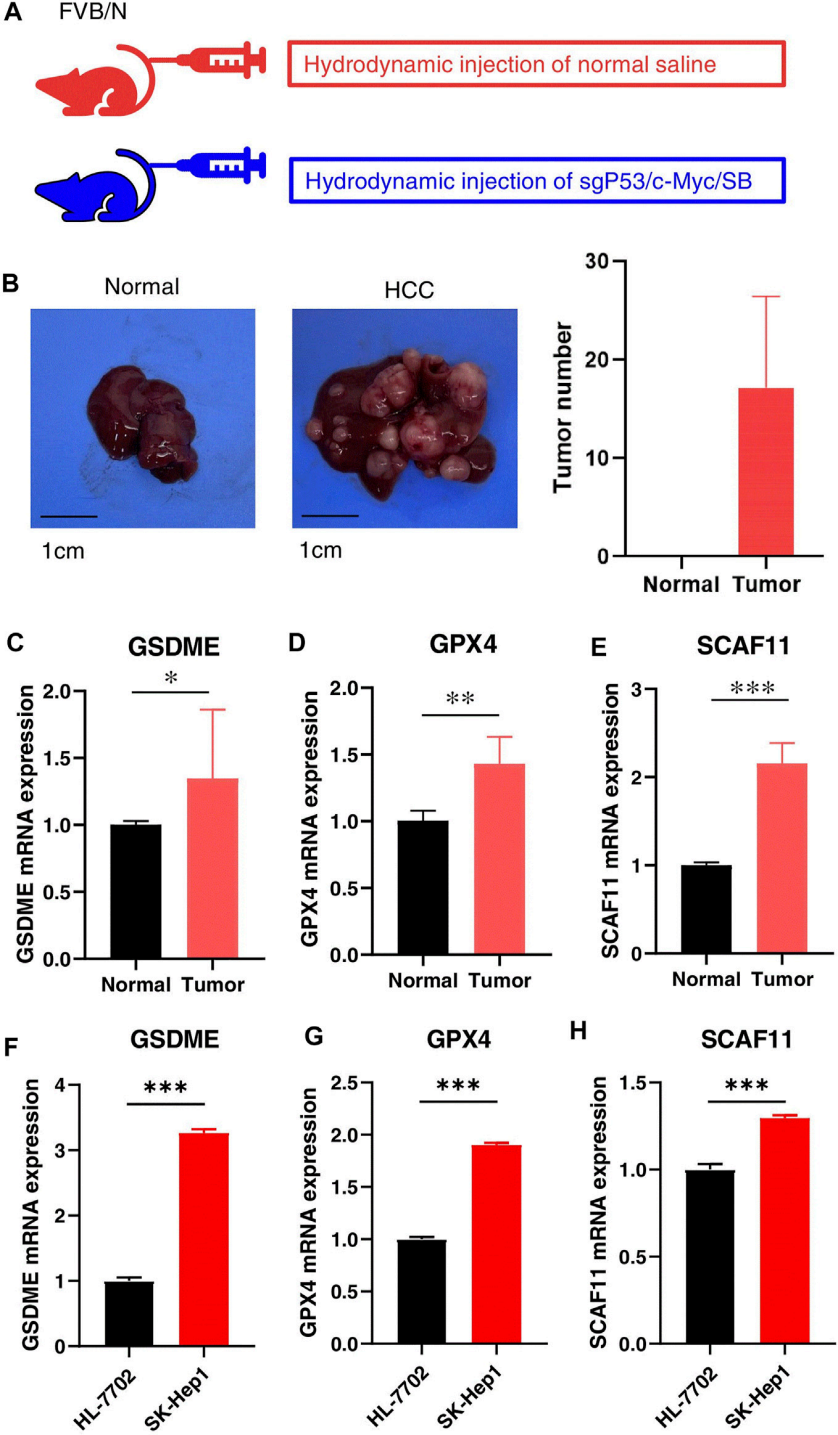
Validation of the independent prognostic genes *in vivo* and *in vitro*. **(A)** HCC Model design. FVB mice were injected with normal saline or sgP53/c-Myc/SB plasmids, respectively. **(B)** Representative liver tissues of normal (left) and HCC mice (middle) and tumor numbers (right). **(C–E)** The mRNA expressions of GSDME, GPX4, and SCAF11 in the normal and HCC livers. **(F–H)** The mRNA expressions of GSDME, GPX4, and SCAF11 in the human normal liver cell HL-7701 and liver cancer cell SK-Hep1. *p < 0.05, **p < 0.01, ***p < 0.001.

We further validated the expression levels of GSDME, GPX4, and SCAF11 in a mouse HCC model, which was constructed by knocking out the *p53* and overexpressing the *myc* in the mouse liver (Figure 10A). The HCC model formed multiple tumors as shown in Figure 10B. As expected, the mouse HCC livers had remarkably increased *GSDME*, *GPX4*, and *SCAF11* mRNA levels compared to the normal livers (Figures 10C–E).

In addition, we confirmed the mRNA levels of these independent prognostic genes in human culture cells. As shown in Figures 10F–H, the HCC cell line SK-Hep1 expressed significantly higher mRNA levels of GSDME, GPX4, and SCAF11 compared to the normal liver cell line HL-7702.

### Drug Sensitivity Analysis

**FIGURE 11:**
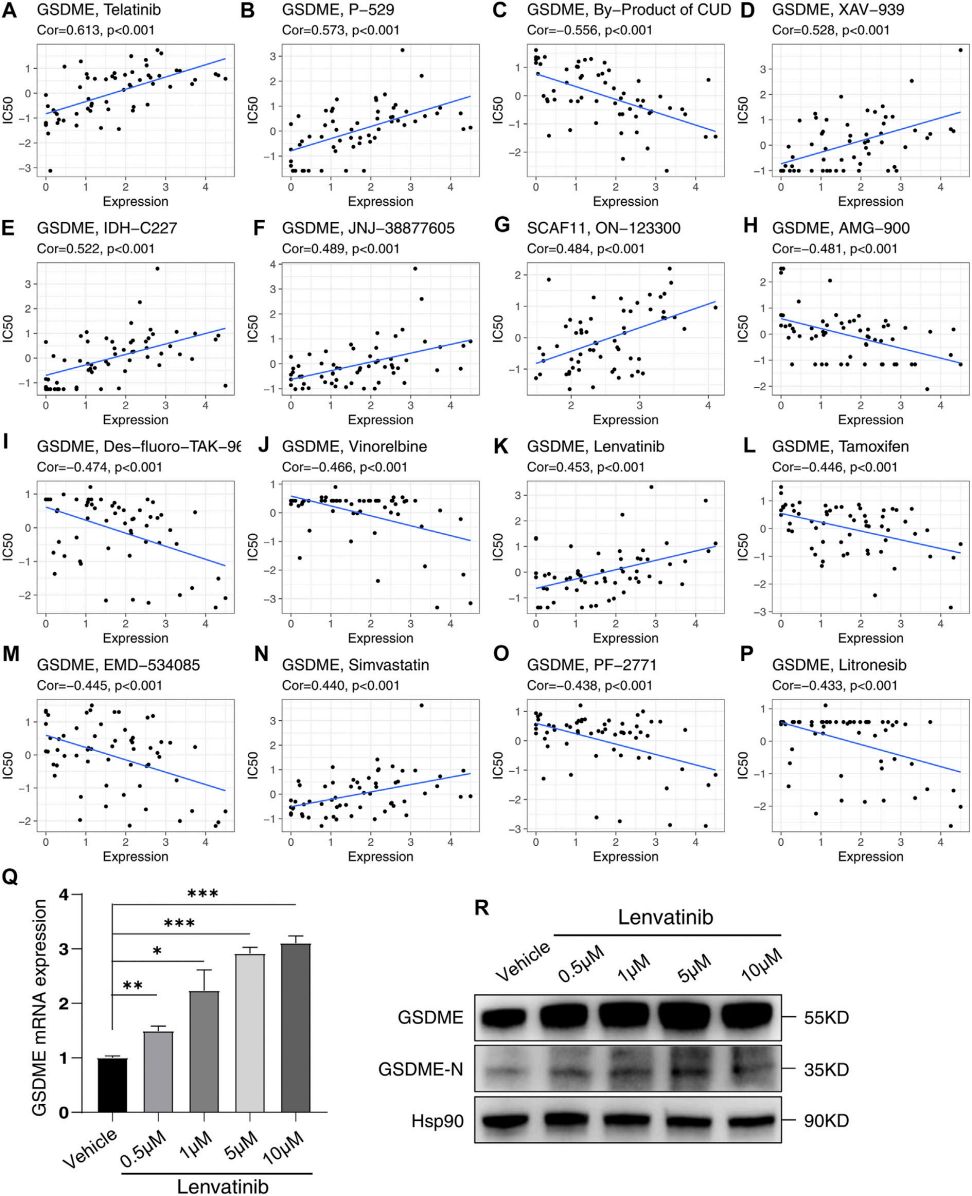
Sensitivity correlation analysis between the independent prognostic genes and drugs based on the CellMiner Database. **(A–P)** Correlation analysis. **(Q)** Relative mRNA expression of GSDME following treatment with Lenvatinib for 48 h in Huh7. **(R)** Analysis of GSDME and GSDME-N terminal in Huh7 treated with Lenvatinib for 48 h by western blotting. *p < 0.05, **p < 0.01, ***p < 0.001.

By analyzing the CellMiner database, the potential drugs were found to be correlated to these independent prognostic genes (Supplementary Table S4). Among the top 16 gene-drug correlations, 15 correlations pointed to the GSDME; the other correlation was SCAF11 (Figure 11). Surprisingly, the HCC drug lenvatinib positively correlated with the expression of GSDME (Cor = 0.453, *p* < 0.001, Figure 11K). To confirm this correlation, we treated HCC cell line Huh7 with Lenvatinib. As was expected, Lenvatinib treatment upregulated both the mRNA and protein levels of GSDME. Besides, Lenvatinib was able to induce the active form of GSDME (GSDME N-terminal).

## Discussion

In recent years, the increasing incidence of HCC has necessitated early diagnosis and prognosis as being incredibly significant for the survival of the patients. At present, the diagnosis and prognosis of HCC patients are mainly based on the pathological evaluation, AJCC TNM, and BCLC stage (Bruix et al., 2016). However, these diagnostic and prognostic methods are not sensitive enough. Therefore, finding efficient diagnostic and prognostic markers is necessary for helping the HCC patients in improving their clinical outcomes. Moreover, the mechanisms underlying the pathogenesis of HCC have not been fully elucidated (Ye J. et al., 2021). To devise precision medicine, improving the prognosis and survival rate of HCC patients, and discovering the molecular mechanisms underlying the development and progression of HCC, accurate and reliable prognostic models should be developed based on the novel biomarkers (Yan et al., 2021). Although the role of pyroptosis in tumors has been gradually explored (Wang et al., 2017; Blasco and Gomis, 2020), it remains unclear mainly in HCC.

This study systematically compared the altered expressions of PRGs in HCC patients and revealed that three genes were significantly downregulated while 23 genes were upregulated. The consensus clustering analysis identified 17 prognostic genes, which helped the HCC patients be divided into two subtypes. Interestingly, the two subtypes were indistinguishable by existing clinical criteria but had significant differential survival rates, proving instrumental for clinical typing. Subsequently, to evaluate the prognosis of the HCC patients, we established an independent prognostic model by performing the LASSO and Cox analysis of the prognosis-related genes, which was well validated in the internal test and external validation cohorts. The model yielded significant survival differences among the patients with different clinical characteristics, except for those with age ≤65 and in stage III-IV. Based on this risk model, a nomogram was drawn to predict the HCC patients’ overall survival. Besides, the high-risk group had a lower immune score and higher expression level of PD-L1 than the low-risk group, indicating the potential differences in the immune function between the two risk groups. Later, the GSEA analysis of the two risk groups revealed that the T cell receptor and B cell receptor signaling pathways were enriched. Furthermore, the ssGSEA analysis suggested that the high-risk group is characterized by the lower levels of immune cell infiltration (B cells, Mast cells, NK cells, and TIL cells) and immune pathways (Cytolytic activity and Type II IFN response). Considering the independent prognostic model was constructed using GSDME, GPX4, and SCAF11, we then validated their differential expressions in the normal and HCC tissues. Using the RNA-seq data from the TCGA and GTEx, and protein expressions from HPA, all the three genes showed higher mRNA and protein levels in the HCC tissues than those in the normal tissues. Furthermore, the expression of these three risk genes was validated in the HCC mouse model and human HCC cell lines. Finally, we analyzed the correlation between FDA-approved drugs and these three targets using CellMiner database. The results revealed that among the 16 most significant correlations, seven drugs showed significantly positive correlations with GSDME, eight drugs conversely correlated with GSDME, and one drug positively correlated with SCAF11. We finally validated the correlation of Lenvatinib and GSDME in mRNA and protein levels.

Since the term “pyroptosis” was raised in 2001 by D’ Souza et al., many studies have focused on this novel pro-inflammatory programmed cell death (Fink and Cookson, 2005). In recent years, pyroptosis has gained increasing prominence in tumor research (Wang et al., 2017; Yu et al., 2019; Wu et al., 2021). However, pyroptosis demonstrates both pro-tumor and anti-tumor functions (Xia et al., 2019). The induction of the cancer cells towards pyroptosis can suppress tumor development, proving to be a promising target for drug discovery. Also, the inflammatory molecules released by the cancer cells undergoing pyroptosis can gradually transform the surrounding normal tissues into cancer cells by changing microenvironments. However, little research has focused on the function and prognosis of multiple PRGs in HCC development (Chu et al., 2016). An independent prognosis model was constructed with three PRGs, including GSDME, GPX4, and SCAF11. GSDME, formerly called DFNA5, was first confirmed in an extended Dutch family with autosomal dominant nonsyndromic hereditary hearing loss (Van Laer et al., 2004). Then, in 2017, it was identified as a new executor of pyroptosis (Wang et al., 2017). GSDME is cleaved by caspase-3 producing the N-terminal fragment (GSDME-NT) that converts the death pathway from apoptosis to pyroptosis. Recently, new studies have recognized GSDME as a conduit for the release of IL-1β to the surrounding microenvironment independent of its capability of triggering cell death (Zhou and Abbott, 2021). Ever since the mechanism of GSDME mediated pyroptosis was revealed, an increasing number of studies have been focused on its role in cancer. In most cancer types, such as breast cancer, colorectal cancer, gastric cancer, and bladder cancer, GSDME has a higher expression level in the normal tissues and is often considered as the tumor suppressor gene by inducing pyroptosis in the cancer cells or by acting on the T lymphocytes through Granzyme B (Xia et al., 2019; De Schutter et al., 2021). Interestingly, ten percent of tumor cells have a higher GSDME expression level (De Schutter et al., 2021). Surprisingly, in this study, the high-risk groups showed increased GSDME levels than the low-risk group. This result demonstrated that the conventional tumor suppressor, GSDME, may act as an oncogene in the HCC microenvironment. It is reasonable to consider GSDME in promoting tumor development. The GSDME -induced pyroptosis can release many IL-1β and other inflammatory factors into the surrounding normal liver cells, inducing the normal cells into the tumor cells in inflammatory conditions (Zhou and Abbott, 2021). Coincidentally, the GSDME-mediated pyroptosis has been verified as the probable key point to respond to the toxic side effects of chemotherapeutic agents (Shen et al., 2021). Besides, some studies have demonstrated that the development of drugs targeting GSDME could be a promising treatment for HCC (Hu et al., 2019; Zhang et al., 2020; Liang et al., 2021; Shangguan et al., 2021). Hence, the function of GSDME in the HCC development and drug treatment requires further investigation. GPX4, a classical selenoprotein belonging to the glutathione peroxidase families, can reduce the membrane peroxidized phospholipids by transferring GSH (Liang et al., 2007), and it is considered to own unique lipid peroxidation inhibitory properties (Dabkowski et al., 2008). Subsequent studies have identified GPX4 as a classical negative regulator of ferroptosis and have roles in various cancers such as clear-cell carcinomas (CCCs), breast cancer, colon cancer (Yang et al., 2014; Seibt et al., 2019; Yang et al., 2020; Lee et al., 2021). Accordingly, recent studies indicated that GPX4 could also promote HCC development via inhibiting ferroptosis (Kim et al., 2020; Alves et al., 2021; Asperti et al., 2021; Chang et al., 2021). For non-cancer liver diseases, we aim to protect live cells from cell death, lipid peroxidation, and ROS release; induction of GPX4 expression was reported to be helpful to inhibit ferroptosis (Mao et al., 2020). However, for HCC, we aim at killing these cancer cells by inducing cell death, so it is beneficial to inhibit GPX4 and then activate ferroptosis (Jin et al., 2020). The latest study by Kang et al. indicated that GPX4 could suppress macrophagic pyroptosis in mice (Guerriero et al., 2015; Zhu et al., 2019). GPX4 knockout activated the lipid peroxidation-dependent caspase-11, which triggered the GSDMD cleavage to induce pyroptosis during polymicrobial sepsis. Here, GPX4 was found to be overexpressed in the HCC tissues, and the high-risk group showed an increased level of GPX4. Given the role of GPX4 in pyroptosis and ferroptosis, developing inhibitors targeting GPX4 might promote the GSDMD-mediated pyroptosis as well as ferroptosis, thus suppressing the survival of the HCC cells and drug resistance. SCAF11 has been previously reported to be involved in pyroptosis (Ye Y. et al., 2021). However, its role in cancer has not been explicitly studied so far. This study showed that the high expression of SCAF11 is related to the poor prognosis in the HCC patients, revealing that the inhibition of SCAF11 should be considered as a target to treat HCC.

Pyroptosis is always accompanied by inflammation and tumor immunity (Tan et al., 2021). However, chronic inflammation exerts essential functions in tumor initiation, progression, and invasion via suppressing the anti-tumor immune responses mediated by the immune cells such as the Natural Killer cells (NK) and M1 macrophages (Raposo et al., 2015). This study found decreased levels of immune cells such as the B cells, NK cells, TIL cells, and Mast cells, and immune pathways such as type II IFN response and cytolytic activity in the high-risk group, indicating that poor prognosis may result due to the decreased levels of anti-tumor immunity. Therefore, promoting anti-tumor immune responses are of great importance for effective clinical therapies.

Analysis of the NCI-60 cell line set in the CellMiner database indicated that the increased levels of the prognosis-related genes are positively correlated to drug resistance, such as Tamoxifen, Vinorelbine, AMG-900, and Litronesib. Notably, an increased level of GSDME is also positively correlated with the sensitivity to Lenvatinib, the first-line drug approved by FDA in 2018 for treating HCC (Yi et al., 2021). In 2019, a study showed that sorafenib, a classic FDA-approved drug for treating HCC, can induce macrophage pyroptosis and promote the NK cell responses against HCC (Hage et al., 2019). This research, combined with the correlation of Lenvatinib and GSDME, made us hypothesize that the mechanisms of Lenvatinib in HCC treatment might also be involved in inducing pyroptosis. We further confirmed this hypothesis by treating Huh7 cells with Lenvatinib, and the results showed that Lenvatinib upregulated the levels of both total GSDME and active GSDME N-terminal. Analyzing the correlation suggested that the pyroptosis-related prognostic genes are promising for anti-tumor drug development.

## Conclusion

Our study revealed that developing HCC is inextricably linked to pyroptosis. Furthermore, the functional analysis, immune microenvironment, and drug correlation analysis established a basis for investigating the role of pyroptosis in the HCC development, determining the prognosis for the HCC patients, and providing clinical treatment. Based on the PRGs, an independent prognostic model was constructed for HCC, predicting the OS of the patients in the TCGA test cohort and ICGC external validation cohort. Moreover, our results confirmed the mRNA expression of three independent prognostic PRGs *in vivo* and *in vitro*. Our work will further assist in understanding the role of pyroptosis in HCC prognosis and drug sensitivity, thereby providing support for precision medicine.

## Data Availability

The original contributions presented in the study are included in the article/Supplementary Material, further inquiries can be directed to the corresponding author.
