# Supplementary material for: Identification and Validation of Pyroptosis-Related Gene Signature to Predict Prognosis and Reveal Immune Infiltration in Hepatocellular Carcinoma
Source: Front Cell Dev Biol. 2021 Nov 8;9:748039. doi: 10.3389/fcell.2021.748039 (PMC8606409; doi:10.3389/fcell.2021.748039)
Supplement: Supplementary file 3 [file Table3.DOCX]

Table S4.

| **Covariates** | **TCGA_Train** | **TCGA_Test** | **P value** |
| --- | --- | --- | --- |
| **Age** | 60.81±13.16 | 58.06±13.76 | 0.051 |
| **Gender** |  |  | 0.855 |
| Male | 126 | 123 |  |
| Female | 60 | 61 |  |
| **Grade** |  |  | 0.931 |
| G1 | 27 | 28 |  |
| G2 | 92 | 85 |  |
| G3 | 60 | 61 |  |
| G4 | 6 | 6 |  |
| Unknow | 1 | 4 |  |
| **Stage** |  |  | 0.247 |
| Stage I | 96 | 75 |  |
| Stage II | 34 | 51 |  |
| Stage III | 44 | 41 |  |
| Stage IV | 2 | 3 |  |
| Unknow | 10 | 14 |  |
| **T** |  |  | 0.405 |
| T1 | 100 | 81 |  |
| T2 | 38 | 55 |  |
| T3 | 39 | 41 |  |
| T4 | 8 | 5 |  |
| Unknow | 1 | 2 |  |
| **N** |  |  | 0.346 |
| N0 | 130 | 122 |  |
| N1 | 3 | 1 |  |
| Unknow | 53 | 61 |  |
| **M** |  |  | 0.953 |
| M0 | 137 | 129 |  |
| M1 | 2 | 2 |  |
| Unknow | 47 | 53 |  |
